# Poorly Differentiated Cutaneous Squamous Cell Carcinoma (cSCC) or Lymphoepithelioma-like Carcinoma of the Skin (LELCS) with Squamous Pearls: A Case Presentation with Emphasis on Histomorphological Features and Classification Debates

**DOI:** 10.3390/life13122265

**Published:** 2023-11-27

**Authors:** Sonia Maniglio, Gerardo Cazzato, Concetta Caporusso, Anna Colagrande, Eleonora Nacchiero, Michele Maruccia, Jacqueline Valerio, Eugenio Maiorano, Leonardo Resta, Andrea Marzullo, Giuseppe Giudice, Giuseppe Ingravallo

**Affiliations:** 1 Section of Molecular Pathology, Department of Precision and Regenerative Medicine and Ionian Area (DiMePRe-J), University of Bari “Aldo Moro”, 70124 Bari, Italy; s.maniglio1@studenti.uniba.it (S.M.); concetta.caporusso@policlinico.ba.it (C.C.); anna.colagrande@gmail.com (A.C.); jacquelinevalerio@tiscali.it (J.V.); eugenio.maiorano@uniba.it (E.M.); leonardo.resta@uniba.it (L.R.); andrea.marzullo@uniba.it (A.M.); giuseppe.ingravallo@uniba.it (G.I.); 2 Section of Plastic Surgery, Department of Emergency and Organ Transplantation (DETO), University of Bari “Aldo Moro”, 70124 Bari, Italy; eleonora.nacchiero@gmail.com (E.N.); michele.maruccia@uniba.it (M.M.); giuseppe.giudice@uniba.it (G.G.)

**Keywords:** LELCS, SCC, poorly differentiated, dermatopathology, lymphocytes, T-cells, B-cells, ISH

## Abstract

Lymphoepithelioma-like carcinoma of the skin (LELCS) is a rare primary skin cancer, with an annual incidence of 1/100,000 and about 85 cases published in the literature. It is considered the cutaneous counterpart of undifferentiated nasopharyngeal carcinoma (UNC, Schmincke–Regaud tumor) but has no association with EBV. We present an interesting case with features of LELCS in a 93-year-old man, right frontal–orbital region, diagnosed histologically and with immunohistochemical features. We also emphasize contrasting morphologic features for correct nosographic classification and address current issues, suggesting potential insights. Finally, we briefly reviewed other cases described in the literature.

## 1. Introduction

Historically, there has always been a heated debate about the correct classification of lymphoepithelioma-like carcinoma of the skin (LELCS) between those who argue that it is a poorly differentiated, uncommon variant of cutaneous squamous cell carcinoma (cSCC) and those who frame LELCS as a separate entity, part of the multitude of existing primary cutaneous neoplasms [1]. Notably, the current major classification systems clearly reflect this classification uncertainty, with the World Health Organization’s (WHO) classification of skin cancers [2] counting LELCS among the uncommon variants of cSCC (code in International Classification of Diseases, ICD, 8082/3) and the Armed Forces Institute of Pathology (AFIP) Atlases of Tumor and non-Tumor Pathology “Nonmelanocytic Tumors of the Skin” recognizing LELCS as an entity in its own right, defined as “rare but distinctive tumor with morphological features reminiscent of undifferentiated nasopharyngeal carcinoma” [3]. LELCS was first described by Swanson S.A. et al., who, in 1988, presented the scientific community with five cases of patients aged 50 to 81 years old with carcinoma that “histologically resembled nasopharyngeal lymphoepithelioma”. In the presented cases, the absolute absence of areas of squamous and/or glandular differentiation and the complete loss of connection with the overlying epidermis were emphasized. Also emphasized was the presence of abundant mucin and an immunophenotype compatible with the epithelial origin of the neoplasm [4]. From there on, many more cases of LELCS have been reported in the literature until a recent review identified more than 60 [5], but there is still an ongoing debate about the correct nosographic classification of this entity. Traditionally, the lack of connection with the overlying epidermis, the cytologic and histologic features, and the absence of clear areas of squamous differentiation have led scholars to assume that this entity was stand-alone, considering it analogous to lymphoepithelioma-like carcinoma of the salivary glands, thymus, tonsils and cervix. On the other hand, if these areas of increased differentiation in the ‘squamous lineage’ were counted among the microscopic features, the tumor was referred to as a poorly differentiated variant of cSCC. In any case, in all but one [6] of the cases published so far, LELCS was negative for Epstein–Barr virus (EBV), unlike the presumed nasopharyngeal counterpart. In this paper, we present a challenging and complexly interpreted case of a lesion with some cytohistologic features of LELCS but with areas of squamous differentiation, in the presence, however, of a complete absence of epidermal connection after repeated cutting. We comment on our features in light of the knowledge gained in the literature and try to interpret our data, suggesting possible and potential and future implications on the recognition of this entity.

## 2. Case Presentation

**Figure 1:**
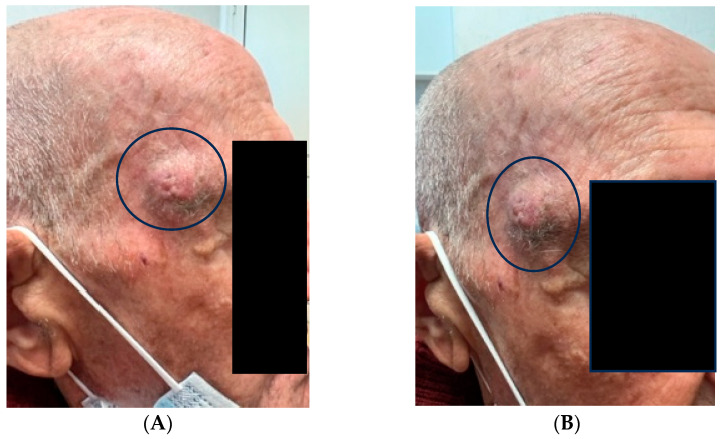
(**A**,**B**) Clinical features of the solitary nodular lesion of 2 cm in diameter (black circles).

A 93-year-old man presented to the referring physician for observation due to the presence of skin lesion of the right temporal region, not better dated, but enlarged over the years (Figure 1A,B). After being referred to the Complex Operative Unit of Plastic and Reconstructive Surgery, surgical excision of the specimen, oriented with two ‘repere’ wires on the upper and lower margin, was performed. The specimen was then sent to the University Pathology Complex Operating Unit, where it underwent macroscopic sampling procedures. The oriented excision of skin and subcutis included a 2 cm, raised, crusted lesion that macroscopically appeared not to involve the lateral margins and apices.

After processing, embedding in paraffin, cutting and setting up the preparation with routine hematoxylin–eosin (H&E) staining, the specimen was observed under a light microscope. Furthermore, immunohistochemical investigations with CD3 (Polyclonal Rabbit anti-Human, Dako-Agilent, Santa Clara, CA, USA) and CD20 (L26, Dako-Agilent) were performed to study the microenvironment of the neoplasm.

## 3. Results

**Figure 2:**
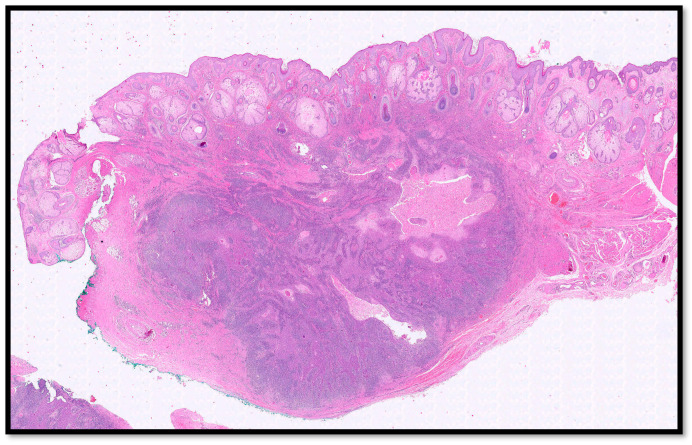
Histological photomicrograph showing tumor nodules composed of two different population of cells, without connection with overlying epidermis (hematoxylin–eosin, 4×). Note the rich density of sebaceous glands typical of the skin of the face.

**Figure 3:**
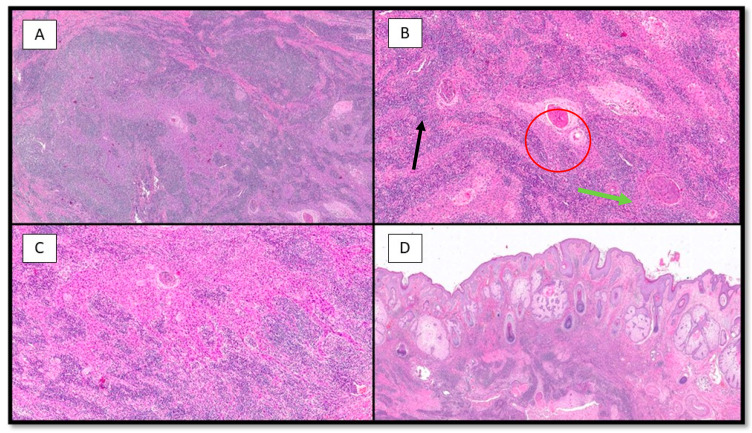
(**A**) Photomicrograph showing a neoplastic lesion that is difficult to discern at this magnification into its two components: epithelial and inflammatory (hematoxylin–eosin, 10×). (**B**) Histopathological micrograph showing the two different components of the lesion: large, epithelioid cells with palely eosinophilic cytoplasm (green arrow) and brisk lymphoplasmocytic inflammatory infiltrate (black arrow). Note the important presence of squamous differentiation (red circle) (hematoxylin–eosin, 20×). (**C**) Low-power magnification of B (hematoxylin–eosin, 20×). (**D**) Photomicrograph showing no connection of the neoplasm with the overlying epidermis (H&E, 4×).

Histologically, the tumor was located predominantly in the dermis, it was lobulated, multinodular with no connection to the epidermis (Figure 2), and consisted of large epithelioid cells with pale, eosinophilic cytoplasm and vesicular, eosinophilic nuclei (Figure 3B,C), and widespread mitotic activity. The tumor cells were arranged in sheets, islands and strands infiltrated by an intense lymphoplasmacytoid inflammatory cell infiltrate (Figure 3A–C). Furthermore, there were islands of squamous differentiation with some neutrophils within.

**Figure 4:**
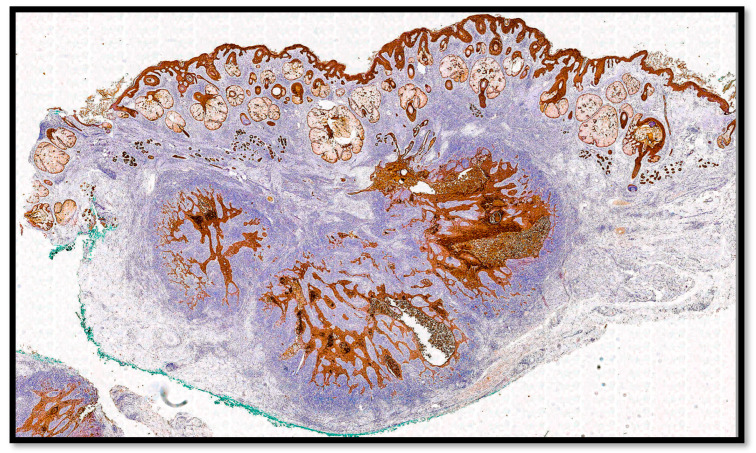
Immunohistochemical preparation for anti-CKAE1-AE3 antibody: note the presence of the diffuse positivity of the neoplasm without any connection with the epidermis (immunohistochemistry for CKAE1-AE3, 4×).

**Figure 5:**
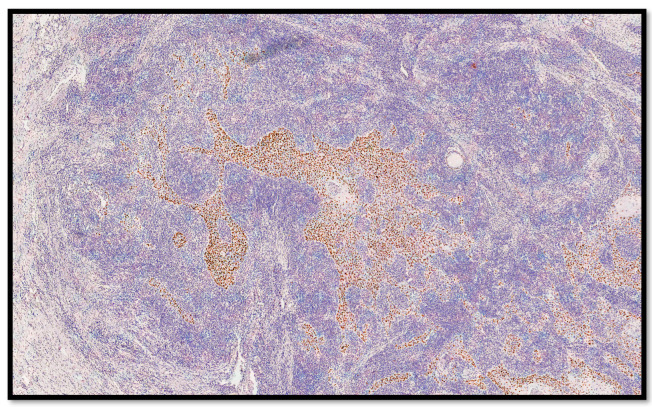
Immunohistochemical preparation for anti-p40 protein: note the diffuse positivity of the strands of the neoplasm (immunohistochemistry for p40, 10×).

**Figure 6:**
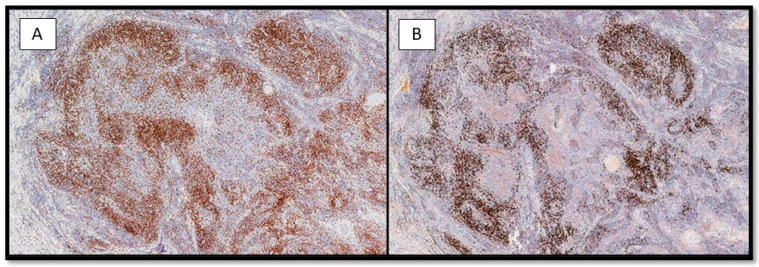
(**A**) Histological photomicrograph showing a rich lymphocytic inflammatory infiltrate composed of CD3-T cells, surrounding the epithelial strands of the neoplasm (immunohistochemistry for CD3, 10×). (**B**) Photomicrograph showing CD20-B cells, surrounding the epithelial strands of the neoplasm in a similar way of the CD3-T cells (immunohistochemistry for CD20, 10×).

From an immunohistochemical point of view, the tumors cells expressed cytokeratin AE1-AE3 (Figure 4) and p40 (Figure 5). Furthermore, the microenvironment was composed of CD3-T cells and CD20-B cells surrounding the sheets, islands and strands of the epithelial neoplasm (Figure 6A,B).

Finally, with the histological and immunohistochemical features, a diagnosis of cutaneous squamous cell carcinoma, lymphoepithelioma-like, was made. The neoplasm extensively infiltrated the subcutis, with a depth of invasion of 7 mm; however, perineural (PNI) and lymphovascular (LVI) invasion could not be appreciated in the examined sections. We staged the lesion as pT1 Nx according to the American Joint Committee on Cancer (AJCC) 8’Edition.

For research purposes, we conducted an In situ Hybridization (ISH) for EBV-encoded RNA (EBER) that demonstrated absolute signal negativity (not shown). Furthermore, we performed a careful work-up of the patient in search of potential primitiveness of the nasopharynx, a completely negative result.

## 4. Discussion

LELCS is a rare neoplasm with an annual incidence of 1 per 100,000 that typically occurs on sun-exposed areas of the head/neck, in elderly individuals, and with no gender predilection [7]. The cSCC is a primary skin tumor with diverse clinical behavior, ranging from indolent to aggressive tumors with considerable metastatic potential [2,8]. Over time, there have been debates about the origin of LELCS, as well as the actual existence of this entity outside of cSCC variants. In this regard, Ho et al., in 2005 [9], hypothesized two theories that could explain the origin of this lesion; according to one interpretation, the origin would be considered epithelial, although in the cases reported in the literature, a connection with the overlying epidermis is never reported, while, on the other hand, since the first description of LELCS [4], it has been thought that it might have an adnexal origin [1,10,11]. In contrast, however, to its nasopharynx counterpart, only one case published so far has demonstrated positivity for EBV [6], with the presence of EBV-DNA in both Real-Time Polymerase Chain Reaction (RT-PCR) and ISH for EBV-encoded RNA (EBER), localized within the nuclei of the tumor cells. All the remaining LELCS cases demonstrated negativity for EBER, suggesting a lack of association. Another study by Kazakov et al. [12] confirmed the absence of, among others, EBV in LELCS samples, suggesting, in the authors’ thinking, that this entity was more likely to be considered a variant of cSCC. Furthermore, Naito R. et al. [13] postulated that LELCS was part of a morphological spectrum rather than an entity in its own right as they drew a parallel on cases of Merkel Cell Carcinoma with a “lymphoepithelioma-like” pattern, and therefore adduced further evidence of possible classification as a poorly differentiated variant of cSCC [13].

Clinically, in the majority of published cases, LELCS was mostly localized on the face, scalp, arms, trunk, and even penis, and the clinical presentation ranged from a single papule up to a nodule. Despite the atypical and alarming histologic features, the reported rates of recurrence and/or metastasis were quite low, except in very rare occurrences [10,11,12,14]. Also, in our case, the lesion presented as a nodule, and after complete excision, 1-year follow-up reported no recurrence/metastasis.

Histologically, LELCS consists of cells with eosinophilic cytoplasm and large vesicular nuclei, surrounded by intense lymphocytic inflammatory infiltrate, consisting of both CD3 T-cells and CD20 B-cells. In our case, the two lymphocyte populations were equivalent, and some authors [15] reported higher rates of infiltration of CD8 T-cells. However, whether these immunophenotypic peculiarities translate into an improved prognosis of LELCS has not yet been clearly demonstrated. The positivity of markers such as CKAE1-AE3, CK5/6 and EMA confirms the epithelial nature of LELCS and an adnexal genesis could also be considered given the frequent presence of foci of adnexal-type differentiation. Regarding this concept, Wick et al. [1] addressed this aspect in their 1991 paper, suggesting that the positivity of CEA (monoclonal), CK18, and CK19 could lead to a potential differentiation into sweat glands. In our case, we preferred to leave the diagnosis “open-ended” since elements not easily agreed upon are present. Indeed, while there was an absence of “attachment” to the overlying skin despite after numerous sections of the neoplasm, the foci of squamous differentiation could lead us to frame this lesion as a variant of cSCC. Considering these different features, we propose that this may be a LELCS with areas of squamous pearls given the absence of any connection with the epidermis.

It is also very important to emphasize the main differential diagnoses to be conducted during the study of LELCS. In particular, one must first rule out a primary lesion in the nasopharynx via imaging (CT scan of the chest) and laryngoscopy with potential swabbing. Only after excluding a secondary location is it possible to consider the entity as a LELCS and consider differential diagnoses such as cutaneous lymphoma and follicular dendritic cell tumors, which, however, do not have an epithelial component [16]. Another differential diagnosis is with MCC, which, however, in addition to cytomorphologic differences (“salt and pepper” chromatin, etc.), will express positivity to neuroendocrine immunohistochemical markers such as Chromogranin A (CgA), Synaptophysin (Syn) and CD56 (NCAM1) [17]. In addition to the differential diagnosis with melanoma, it is crucial to distinguish LELCS from cutaneous lymphadenoma (CL), which lacks malignant neoplasm-like attitudes (ulceration, destruction of pre-existing adnexal structures, infiltration of the deep dermis and/or subcutis) and has an abundant stromal component, which is scarce or absent in the case of LELCS. Finally, it is important to consider that plasma cells are present in the inflammatory infiltrate of LELCS in a certain number [18,19,20].

Scott K. et al. reported three interesting cases of LELC in the vagina (2) and anal canal (1) finding a total absence of EBV and presence of high-risk Human Papilloma Virus (HPV) 16, suggesting the possibility of such infection in lesions of the gynecological and anal tract [21]. Furthermore, still regarding differential diagnosis, it is important to cite the only case described in the literature (by Gebauer N. et al. [22]) up to now of a collision tumor consisting of a LELCS and a primary cutaneous lymphoma of the marginal zone (PCMZL) in a 75-year-old patient on the left cheek. In this particular case, the authors described how a possible mechanism of PCMZL etiopathogenesis could reside in the chronic inflammatory stimulus possibly exerted by the epithelial strands of the LELCS; furthermore, in the case presented, no positivity for Borrelia burgdorferi DNA or other infectious agents was found, which are hypothesized to sometimes be the cause of PCMZL. Proposing, therefore, an analogy with what happens in the case of MALT-lymphoma (chronic inflammatory stimuli and/or Helicobacter Pylorii infection), in the case of LELCS, a potential clonal evolution of the lymphocytic infiltrate could also be mentioned to the point of determining the tumor collision described. In our case, however, the lymphocytic component was typical and monomorphic, without features of concern.

It should be mentioned how the correct biopsy procedure is essential to reach a correct diagnosis of LELCS; in particular, as in the case presented in [13], if a superficial biopsy is conducted, it is possible that the extensive lymphocytic inflammatory infiltrate masks the underlying neoplasm, leading the pathologist to diagnose a reactive inflammatory process.

In terms of treatment options, many treatment techniques have been proposed, including electrocoagulation and curettage, Mohs micrographic surgery, wide excision, radiation therapy and chemotherapy. Despite all these alternatives, wide excision with at least 1 cm free remains the treatment of choice [23,24], and for recurrences or patients with lymph node/distal metastasis or perineural invasion, radiation and chemotherapy can be employed [5].

From the point of view of prognosis, LELCS, despite the presence of alarming histological and cytological characteristics, does not present high rates of metastasis, but a certain possibility of local recurrence [1,25].

## 5. Conclusions

LELCS is a very rare entity that has been debated for decades regarding its correct origin and, therefore, nosographic classification. Despite the increase in the number of cases published in the literature, there is still no definitive pathologic criteria to definitively classify LELCS as a stand-alone entity or as a variant of cSCC. Despite the histologic appearance of “aggressive” neoplasm, the reported recurrence and/or metastatic rate is low, and the prognosis of LELCS is quite good. In our case, conflicting histopathological criteria paved the way for different kinds of interpretations, while considering that perhaps classification as LELCS with areas of differentiation in the squamous sense may be the best diagnostic option. As further cases are published, we will better understand the role of the lymphocytic inflammatory infiltrate, whichn is an essential criterion for the diagnosis of LELCS.

## Data Availability

Data are contained within the article.
